# Rethinking the Connections between Ecosystem Services, Pollinators, Pollution, and Health: Focus on Air Pollution and Its Impacts

**DOI:** 10.3390/ijerph19052997

**Published:** 2022-03-04

**Authors:** Manuela Plutino, Elisa Bianchetto, Alessandra Durazzo, Massimo Lucarini, Luigi Lucini, Ilaria Negri

**Affiliations:** 1 CREA-Research Centre for Forestry and Wood, Viale Santa Margherita 80, 05025 Arezzo, Italy; manuela.plutino@crea.gov.it; 2 CREA-Research Centre for Agriculture and Environment, Via di Lanciola, 12/A, 50125 Florence, Italy; elisa.bianchetto@crea.gov.it; 3 CREA-Research Centre for Food and Nutrition, Via Ardeatina 546, 00178 Rome, Italy; massimo.lucarini@crea.gov.it; 4 Department for Sustainable Food Process, Università Cattolica del Sacro Cuore, Via Emilia Parmense 84, 29122 Piacenza, Italy; luigi.lucini@unicatt.it; 5 Department of Sustainable Crop Production (DI.PRO.VE.S.), Università Cattolica del Sacro Cuore, Via Emilia Parmense 84, 29122 Piacenza, Italy; ilaria.negri@unicatt.it

**Keywords:** ecosystem services, pollinators, pollution, forest ecology, airborne particulate material, health impact

## Abstract

Ecosystems provide many services that are essential for human activities and for our well-being. Many regulation services are interconnected and are fundamental in mitigating and hindering the negative effects of several phenomena such as pollution. Pollution, in particular airborne particulate matter (PM), represents an important risk to human health. This perspective aims at providing a current framework that relates ecosystem services, regulating services, pollination, and human health, with particular regards to pollution and its impacts. A quantitative literature analysis on the topic has been adopted. The health repercussions of problems related to ecosystem services, with a focus on the effects of atmospheric particulate matter, have been highlighted in the work throughout a case study. In polluted environments, pollinators are severely exposed to airborne PM, which adheres to the insect body hairs and can be ingested through contaminated food resources, i.e., pollen and honey. This poses a serious risk for the health of pollinators with consequences on the pollination service and, ultimately, for human health.

## 1. Introduction

Ecosystems provide many services essential for human well-being. Ecosystem services support the economic growth and livelihoods of human populations. Since the 1980s, many authors have described the benefits for people derived from nature [1,2,3,4,5]. Acharya et al. [6] state in a review that over 80% of studies on ecosystem services briefly addressed the multiple functions of ecosystems by focusing mainly on regulatory services (pollination, defense by pollution, climate regulation, etc.). The authors also highlighted that the publications on these regulation services were significantly increased, in particular towards the assessment of pollination and pollution reduction.

According to Osman & Shebl [7], the pollination of crops is one of the essential ecosystem services related to human food production. Pollination services derive mainly from the activity of wild species, such as solitary bees or managed honeybees. Pollinators represent key components for providing other ecosystem services such as biodiversity as well [8]. For more than two decades, the decline in pollinators has been described in many countries, and some factors contributing to the stress for pollinators are as follows: habitat loss, urbanization, increase in pests and diseases, uncontrolled use of pesticides, and climate change [9,10]. Climate change is one of the major problems for pollinator biodiversity, as it causes loss of agricultural productivity and negatively affects food security [11].

Ecosystem services and human well-being are strongly linked to biodiversity. Biodiversity is known for producing food, guaranteeing nutritional security, and providing medicinal essences helpful in treating various diseases [12,13]. Biodiversity and, consequently, ecosystems are affected by several threats such as habitat loss, environmental pollution, and climate change [14]. Pollution, in particular, airborne particulate matter (PM), constitutes an important risk to human health. Vegetation is a useful instrument to decrease PM concentration [15,16]. Trees improve inhabitants’ environmental and life quality by acting as filters, especially for people living in urban areas [17,18]. The impact of airborne PM on human health has been well studied, whereas the impacts on ecosystems and its components, including pollinators, are still largely unknown, even if it would directly affect our health and well-being.

The goal of this paper is to link the purposes/uses of ecosystem services to the effects on public health by studying the impacts of atmospheric particulate matter on bees. The innovation of this perspective is to provide a complete and current view of the regulation services offered by ecosystems and linked to specific aspects usually treated individually not only through careful bibliographic research but also through the treatment of a case study. Pollination and health effects on both humans and pollinators, especially bees, are dealt in connection with specific and parallel issues on ecosystems, such as the effects of atmospheric particulate matter.

## 2. Ecosystem Services, Pollinators, and Biodiversity

The linkage between ecosystem services, pollinators, and health is explored throughout the following topics: (i) literature quantitative research analysis; (ii) conceptualization and classification of ecosystem services; and (iii) pollination, a regulating service for the conservation of biodiversity.

### 2.1. Literature Quantitative Research Analysis

A current and comprehensive analysis of the ecosystem service and pollinator/pollination relationships present in literature is here given. On 23 October 2021, the Scopus database was selected to mine literature in order to retrieve ecosystem service and pollinator/pollination relationship publications. The search string (“Ecosystem service*” AND “Pollinator*” OR “Pollination*”) was used to extract bibliometric data from the Scopus online database (https://www.scopus.com/home.uri, accessed on 23 October 2021) and bibliographic data, i.e., publication year, publication count, document type, countries/territories of origin, and institutions, were recorded. The functions of the Scopus online platform named “Analyze” and “Create Citation Report” were utilized for carrying out basic analyses, whereas, for further bibliometric analyses and additional processing, the “full records and cited references” were exported to VOSviewer software (version 1.6.16, www.vosviewer.com accessed on 23 October 2021). The terms/words used in the titles and abstracts of publications were analyzed by the VOSviewer software (v.1.6.16, 2020), and the paragraphs were broken down into words and phrases by linking them with the citation data of the publications to visualize a bubble map as an expression of the results [19,20,21].

**Figure 1:**
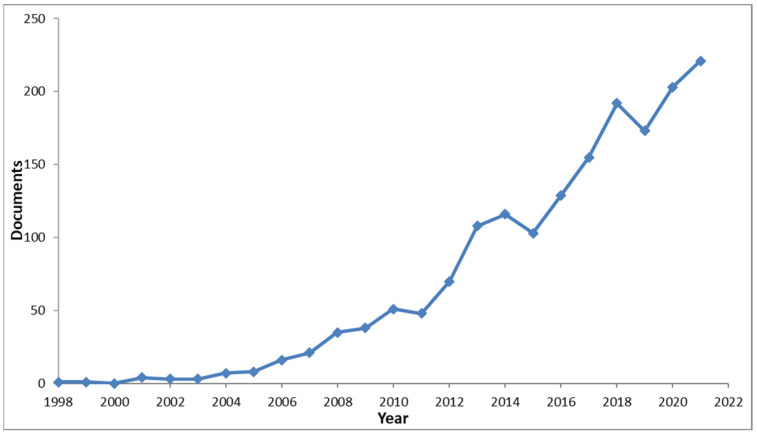
Publication trends (1998–2021) of the ecosystem service and pollinator/pollination relationship search. (Based on data retrieved from Scopus database).

In the range of years from 1998 to 2022, 1710 publications were retrieved by the literature search, and 37.948 documents were collectively cited. The publications trends (1998–2021) are reported in Figure 1. The oldest publications are represented by: (i) a work of Kearns et al. [22] published in 1998 on the conservation of plant–pollinator interactions as endangered mutualisms and (ii) a work of Dong [23] published in 1999 that delineated ecosystem services as the biological conditions and ecological supports necessary for the development of human societies.

**Figure 2:**
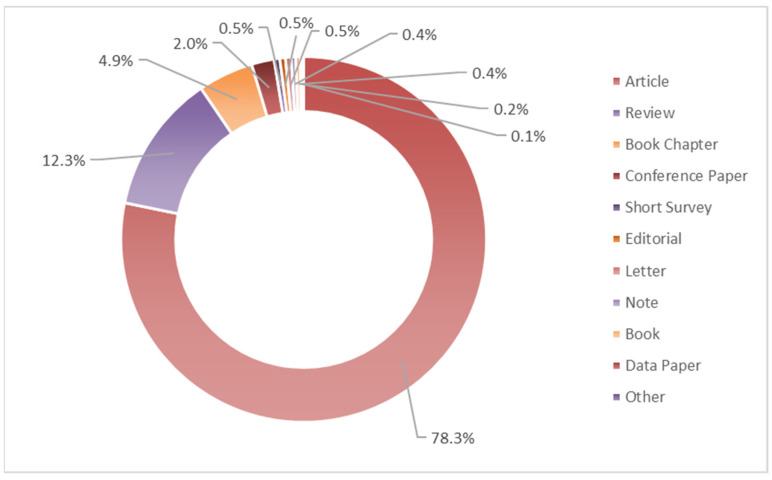
Distribution of documents by type concerning the ecosystem service and pollinator/pollination relationships publications. (Based on data from Scopus).

Figure 2 reported the distribution of documents by type. It includes mainly “Article” for the 78.3%, followed by “Review” (12.3%) and “Book Chapter” (4.9%). The most cited “Book” document (254 times) was published in 2009 and is entitled *Biodiversity, ecosystem functioning, and human wellbeing: an ecological and economic perspective* [24]. For the “Editorial” category, the most cited document is addressed on forest biodiversity, ecosystem functioning, and the provision of ecosystem services [25], whereas the most recent one is published by Dalsgaard [26] in 2020 in *Diversity* on land use and climate impacts on plant–pollinator interactions and pollination services.

**Figure 3:**
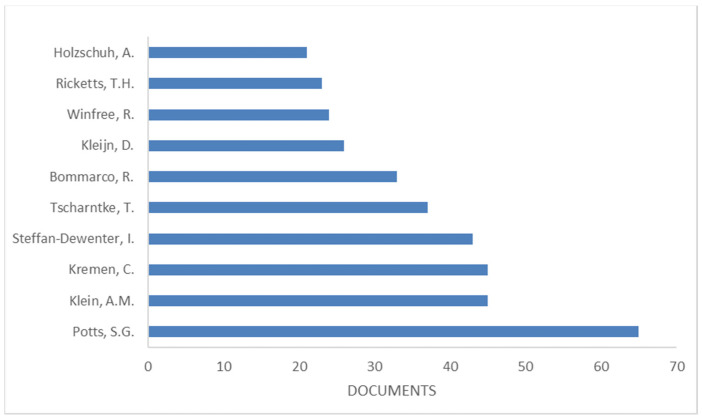
The most productive authors (Based on data from Scopus).

The top productive “Authors” are reported in Figure 3. Pott, S.G. was the most productive author with 65 documents. The following “Short Survey” documents were included: one on the potential impacts of insecticides on the life-history traits of bees and the consequences for pollination [27], and the other one was on the preference valuation of the non-market benefits of pollination services in the UK [28]. Among the “Letter” category, it is worth mentioning the document of Martin et al. [29] that describes the interplay of landscape composition and the configuration of new pathways to manage functional biodiversity and agroecosystem services across Europe by underlining how the enhancing edge density in European agroecosystems can promote functional biodiversity and yield-enhancing ecosystem services.

Another “Letter” document is focused on how mass-flowering crops can represent a tool to dilute pollinator abundance in agricultural landscapes across Europe [30].

Moreover, the most recent document reported for this author investigated the field boundary features that can stabilize bee populations and pollinate mass-flowering crops in rotational systems [31].

**Figure 4:**
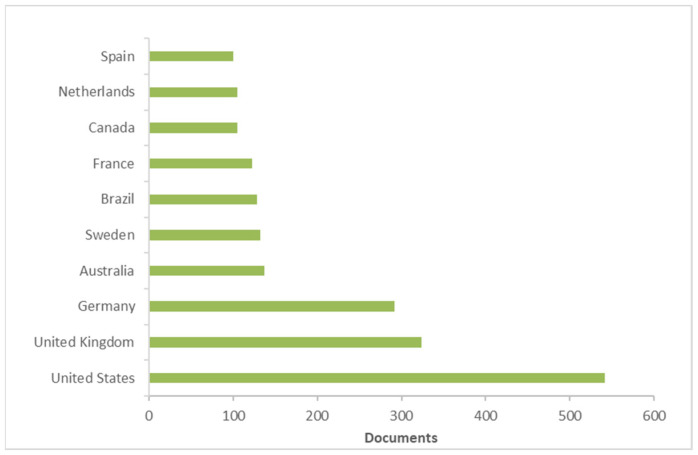
Most productive countries/territories. (Based on data from Scopus).

**Figure 5:**
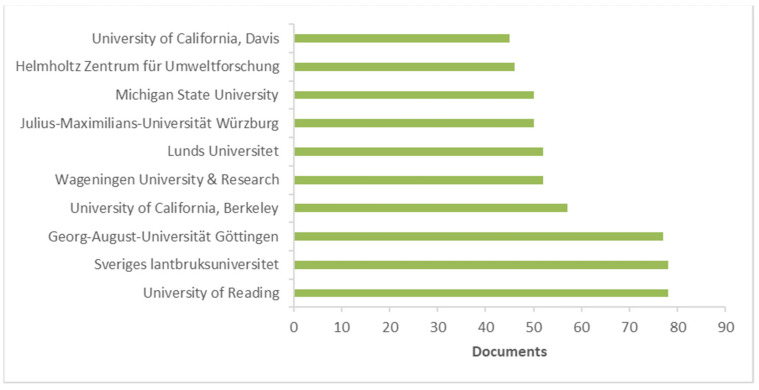
Most productive institutions. (Based on data from Scopus).

Figure 4 and Figure 5 report the most productive countries/territories and institutions, respectively. Regarding countries/territories, the United States (n = 541) was the most productive country, followed by the United Kingdom (n = 324) and Germany (n = 292).

The main keywords covered for documents reported for the United States are: pollination, ecosystem service/s, biodiversity, pollinator, *Apoida*, bee, and animal/s. Its most cited article is on the effect of plastic low tunnels on natural enemies and pollinators in New York strawberry [32], whereas the most cited review is focused on global trends regarding the number and diversity of managed pollinator species by highlighting the need to prioritize biodiversity-friendly measures (hence maintaining the diversity of native pollinator species and providing ecosystem resilience to future environmental changes) [33].

The most productive institutions were the University of Reading and Sveriges lantbruksuniversitet with 78 documents each. All the Top 10 institutions contributed with at least 45 publications or more. For the University of Reading, the most recent document is focused on productivity, biodiversity trade-offs, and farm income in agroforestry versus an arable system and concluded how a diversified farming system could improve farm income, but support from grants would reduce the initial negative cash flow [34]. For Sveriges lantbruksuniversitet, the most recent document is a review by Baho et al. [35] on microplastics in terrestrial ecosystems by moving beyond the state-of-the-art to minimize the risk of ecological surprise.

**Figure 6:**
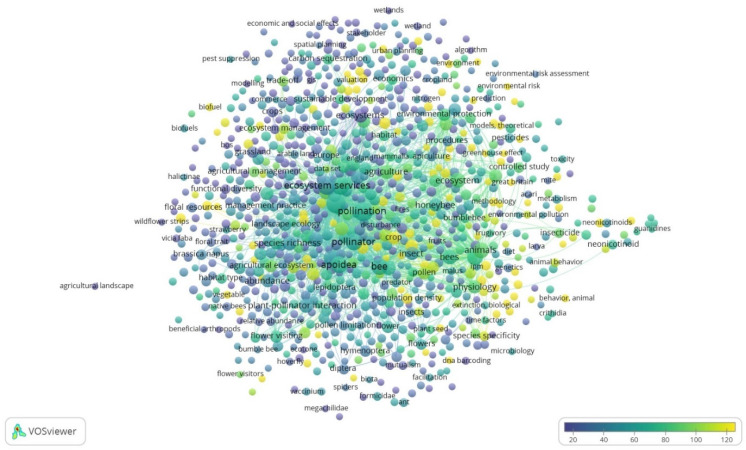
Term map for search ecosystem service and pollinator/pollination publications. Bubble size indicates the number of publications. Bubble color represents the citations per publication (CPP). Two bubbles are closer to each other if the terms co-appeared more frequently. (Based on data from Scopus and elaborated by VOSviewer software).

In total, 943 terms were identified from the quantitative literature research on 1710 publications, and they are visualized as a term map in Figure 6. The top recurring terms on the ecosystems service and pollinator/pollination research are: pollination, ecosystem service/s, biodiversity, bee/s, pollinator, animal/s, *apoida*, and *hexapoda*.

Narrowing the search under the perspective of biodiversity, sustainability, and health relationship, the following search string returned eight publications: TITLE-ABS-KEY: (“pollinator*” OR “pollination*” AND “ecosystem service*” AND “biodiversity*” AND “sustainability*” AND “health*”. It is worth mentioning the book chapter on ecological intensification as managing biocomplexity and biodiversity in agriculture through pollinators, pollination, and deploying biocontrol agents against crop and pollinator diseases, pests, and parasites [36]. Another book chapter by [37] described essential approaches for sustainable and climate-smart land use in agroforestry field, such as the expansion of the species characterizations, the use of underutilized species, the intensification of using beneficial soil organisms for soil and plant health, maximizing resource use efficiency, minimizing pest incidence, and the creation of climate-smart and pest-suppressive landscapes by improving the valuation of environmental services. Among “Article” documents, the following ones are reported: -investing in the transition to sustainable agriculture [38]; -organic agriculture supports biodiversity and sustainable food production [39]; -prospects from agroecology and industrial ecology for animal production in the 21st century [40].

Among the “Review” category, two documents were reported: One described the integration of biodiversity and conservation with modern agricultural landscapes [41], and the other one focused on the human health impacts of ecosystem alteration [42].

### 2.2. Ecosystem Services: Concept and Classification

The concept and classification of ecosystem services is given here. Some authors define ecosystems’ biological, habitat, and system properties as “ecosystem functions” [2]. The same authors describe ecosystem services as “the benefits that human populations derive, directly or indirectly, from ecosystem functions. In particular, ecosystem goods are, for example, food or raw materials, while ecosystem services are, for example, the processes of decomposition and recycling of organic matter”.

Daily [3] defined ecosystem services as “the conditions and processes by which natural ecosystems support and satisfy human life”. For the project Millennium Ecosystem Assessment [43], ecosystem services represent all the benefits that humanity derives from the natural world.

The Millennium Ecosystem Assessment (acronym MA) started in 2001. The MA’s objective was to assess the consequences of changes in ecosystems on human well-being and the scientific basis for actions needed to improve conservation and the sustainable use of such systems and their contribution to human well-being. MA involved over 1360 experts around the world. The results of the MA were published in 2005 in five technical volumes and six summary reports. The state-of-the-art on the condition of the world’s ecosystems was formulated and the services they offer (such as clean water, food, forest products, flood control, and natural resources) and options for restoring, conserving, or improving the sustainable use of ecosystems were delineated. Future scenarios were also developed based on the trend of observed changes in ecosystems.

Recently, Costanza et al. [44] defined ecosystem services as the relative contribution of natural capital to human well-being in the form of material products, i.e., procurement services for timber, food, and intangible products, i.e., habitats, erosion prevention, and aesthetic and recreational value [3].

Nowadays, some authors [45,46] classify ecosystem services into four categories:

1. Provisioning services: material or energy products obtained from wild and cultivated plant species such as food, water, wood, fuels, non-wood products, etc., and medicinal products.
2. Regulating services: the benefits obtained from the regulation of ecosystem processes, such as climate regulation through carbon sequestration, air quality, and the mitigation of extreme events, the prevention of erosion and fires, pollination, etc.
3. Cultural services: non-material and perceptive benefits such as aesthetic and recreational value, spiritual, cognitive enrichment, etc.
4. Support services: these are general services necessary for the production of other SEs. They can include primary production, nutrient cycling, soil enrichment, creating or maintaining habitats for various species, etc.

Currently, Baskent [47] compares ecosystem services to “baskets” of benefits that ecosystems provide to the people who can benefit from them.

**Figure 7:**
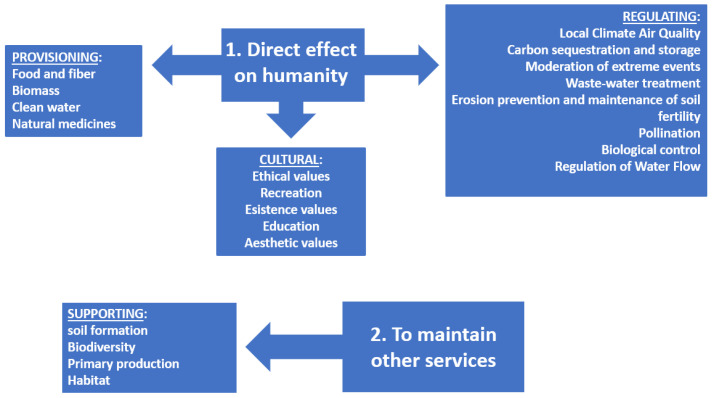
Ecosystem services: definition of ecosystem services’ objective (direct effect on humanity—to maintain other services), classification of them in four categories (provisioning, regulating, cultural, and supporting), and list of some of the services included in each category.

Some services, such as soil erosion control, can be categorized as support and regulating services, depending on the time scale considered and their short-term impact on humanity (Figure 7).

Like all ecosystems, grasslands, forests, and agricultural land are capable of generating ecosystem services. They contribute to human well-being in several ways by ensuring the primary material for good life and health [43,48] and make an important contribution to the elements of human well-being [49,50]. For example, regulating services contribute significantly to human health throughout climate regulation, air quality, disease control, and safety through water regulation, and the regulation of natural hazards such as fires [51]. In Table 1, the role of trees in regulating services is reported by the FAO website. Cultural services contribute to strengthening social relations [52].

Extensive research has been conducted to clarify the link between biodiversity, functioning, and ecosystem services [47]. Numerous experimental evidence indicates that the positive effects of biodiversity on most ecosystem services, particularly regulating and supporting [47].

### 2.3. Pollination: A Regulating Ecosystem Service

The pollination service of crops by insects represents a necessary ecosystem service. Klein et al. [53] reported that fruit and vegetable production of 87 out of 115 species depends on animal pollination. In 2009, Gallai et al. [54] estimated that the value of insect pollination was 153 billion, 9.5% of the world agricultural production used for human food. Pollinators perform an ecosystem service of enormous importance for nature and man. Specific relationships are established between the plant and the pollinator, resulting from long co-evolutionary processes between angiosperms and insects.

In 2012, Thakur [55], in his review, stated that bees pollinate 16% of all plant species and that equates to 0.25 million and that, worldwide, 90% of the food supply is provided by 82 products derived from plant species, of which 63 (70%) are pollinated by bees. In fact, bees are the most valuable pollinators in agriculture.

The same author affirmed that there are many species of bees, and they have great biodiversity and are known worldwide: carpenter bees, bumblebees, megachylids, halictidae, specidae, andrenidae, etc. The native species seem more efficient because they are operational both at the beginning of the day and in the following part. They are able to accumulate both pollen and nectar by allowing hum pollination rather than contact pollination and by favouring honey bees to move between flowers. Another good thing about native species is that they can integrate other species if colonies are difficult to acquire. It is essential to characterize the biodiversity of honey bees and other pollinators through product analysis.

In Table 2, some initiatives to increase the wild bee populations and implement/promote the conservation and sustainable use of pollinators are reported, established under the convention on biological diversity that envisages the following initiatives at least in the eastern half of the United States [56].

Plantings, on which wild bees may forage or reproduce, are also made and protected from fires, floods, overgrazing, or insecticide exposure. Otherwise, little is known about the manipulation of the thousands of other species of wild bees.

The pollination service carried out by bees is essential for agricultural activities. Bees, however, also perform other no less important services. One of these is their role as environmental bioindicators. Until a few decades ago, the great part of the research on pollinators as bioindicators was carried out at the population level and focused primarily on bees [57]. For some decades, an important bioindicator of ecosystemic stress has been the value of pollinator assemblies. The size of pollinator populations represents one of the most important variables for plant reproduction, especially throughout agricultural production, this aspect of their role as bioindicators is primarily reviewed [58].

The role of bees as a bioindicator has become increasingly fundamental, especially as the negative effects that air pollutants bring to ecosystems increase. Bees are affected by pollutants and, for this reason, they have been studied as bioindicators to monitor pollutants. Honey, pollen, or both can be contaminated by various pollutants, of which traces remain detectable through specific analytical instruments [59].

One of the most striking early examples of the role played by bees refers to the Chernobyl disaster. In April 1987, several studies measured the number of radioisotopes in honey and pollen and demonstrated the effective use of bee colonies as local, regional, and global environmental quality samplers [60,61]. Other studies from the same period showed the possibility of measuring the presence of fluorides [62] and organic compounds (e.g., PCBs and pesticides) [63,64] in floral nectar, pollen, and on the body of bees. Bees now represent excellent bioindicators in natural, agricultural, industrial, and urban environments [65,66,67,68].

After more than 40 years after these cited studies, nowadays, honey bees represent important bioindicators that should be used, considering how they well meet the criteria for selecting bioorganisms for the evaluation of environmental pollutants. Current studies analyzed the levels of Manganese (Mn), Cadmium (Cd), Lead (Pb), Nickel (Ni), and Chromium (Cr) in fresh bee and bee honey samples collected in areas with varying degrees of pollution interior of the State of Oyo (Nigeria) to determine the potential of bees and honey bees as bioindicators of environmental pollution by heavy metals. The study confirmed the validity of the levels of pollutants resulting from the samples taken from the bioindicators [69]. Current works demonstrate an innovative contribution of the honey bee as a bioindicator of airborne PM, a ubiquitous pollutant known to kill about 5 million people a year and harm billions more.

In the following paragraphs, we will focus on pollutant PM characteristics and the impact of its exposure on human health, the innovative contribution of the honey bee to assess airborne PM pollution annd the impact of PM exposure on the bee health.

## 3. Airborne Particulate Matter (PM) and Characteristics and Effects on Human Health and Pollinators: A Case of Study

### 3.1. Main Features of Airborne PM and Impact of Its Exposure on Human Health

Particulate matter (PM) is a mixture of airborne chemical compounds, commonly classified by size. Particles less than 100 μm in diameter are referred to as total suspended particulate and include PM10 (up to 10 μm), PM2.5 (up to 2.5 μm), PM1 (up to 1 μm), and ultrafine PM (PM0.1).

Once inhaled, PM10 can penetrate the respiratory tract below the larynx, while PM2.5 can penetrate the gas exchange region where the ultrafine fraction can also cross the alveolar epithelium [70]. PM can be directly emitted as primary compounds or may derive from the chemical transformation and condensation of gaseous pollutants, including nitrogen and sulfur oxides, ammonia, and volatile organic compounds. Among the primary emission sources are the natural erosion of rocks and soils, forest fires, marine aerosol, and volcanic eruptions. In urban areas, major sources of dusts include industrial processes, combustion of wood, and fossil fuels, incineration of wastes, vehicular traffic, and agricultural operations [70].

The human health hazard associated with PM exposure is universally acknowledged. PM exposure is responsible for both short-term and long-term health effects [71,72], with the finer fraction being proportionately more toxic at low-dose exposure [73].

Long thought to primarily harm the respiratory system through induction of allergic responses, asthma, cardiopulmonary diseases, and lung cancer, exposure to airborne PM is currently associated with a wide range of gastrointestinal disorders. Indeed, oral exposure to PM may frequently occur through swallowing or by ingesting contaminated food [74,75,76].

Even if the potential to elicit the adverse biological effects is intimately linked to the size, morphology, and chemical composition of PM, health-based limits of PM exposure currently set by environmental organizations only refer to the average mass concentration of “generic” PM10 and PM2.5 sampled daily or annually by ground monitoring stations, with no need for further specific characterization (e.g., Directive 2008/50/EC of the European Parliament and the Council of 21st May 2008 on ambient air quality and cleaner air for Europe).

However, many toxicological studies demonstrate that the shape of the particle is critical for its interactions with biological systems, independently of its chemical composition. For example, this is the case of needle-like PM for which the “fiber paradigm” has been developed. This paradigm states that the biological mechanisms of particle clearance are typically affected by the length, diameter, and biopersistence of fibers [77,78], and therefore, exposure to fibres of different composition, from naturally occurring silicate minerals belonging to the serpentine and amphibole groups (commonly known as asbestos) to vitreous and ceramic fibres, leads to similar adverse effects on living organisms [77,79].

Regarding particle size, the ultrafine fraction is also of much concern. PM0.1 stays airborne longer, and exposure may be prolonged. Ultrafine particles can also easily gain access to alveoli, where they can cross epithelial barriers and enter blood circulation [80,81,82]. Nasal PM0.1 may also enter the brain directly via the olfactory bulb [82]. According to some authors, metal-based PM0.1 promotes DNA damage via oxidative stress, epigenetic alterations with consequences on gene expression [83,84,85], and even neurological disorders such as Alzheimer’s and Parkinson’s diseases [80,81].

Studies on the application of nanotechnology for medical purposes demonstrate that the shape of nanosized PM plays a crucial role in determining the biological interactions, including endothelial transport, phagocytosis, and interaction with blood vessels [78]. For example, round-shaped PM0.1 tends to stay at the center of the blood vessel and thus circulates more easily inside the body than rods, discs, hemispheres, or ellipsoids [78,86], which tends to accumulate towards the vessel wall, where they may bind to wall receptors or cross the endothelium. In addition, the cellular uptake of spherical PM0.1 occurs more quickly than particles with different shapes [86].

### 3.2. The Contribution of the Honey Bee to Assess Airborne PM Pollution

Since exposure to airborne dusts is ubiquitous and linked with several adverse health effects, mainly due to its size and chemical composition, there is a strong need to develop efficient monitoring techniques that can also provide information on the nature and chemical-physical characteristics of airborne PM. In this regard, the use of the honey bees to assess airborne PM pollutants may add valuable contributions to the data provided by fixed air sampling systems commonly employed for monitoring airborne PM.

**Figure 8:**
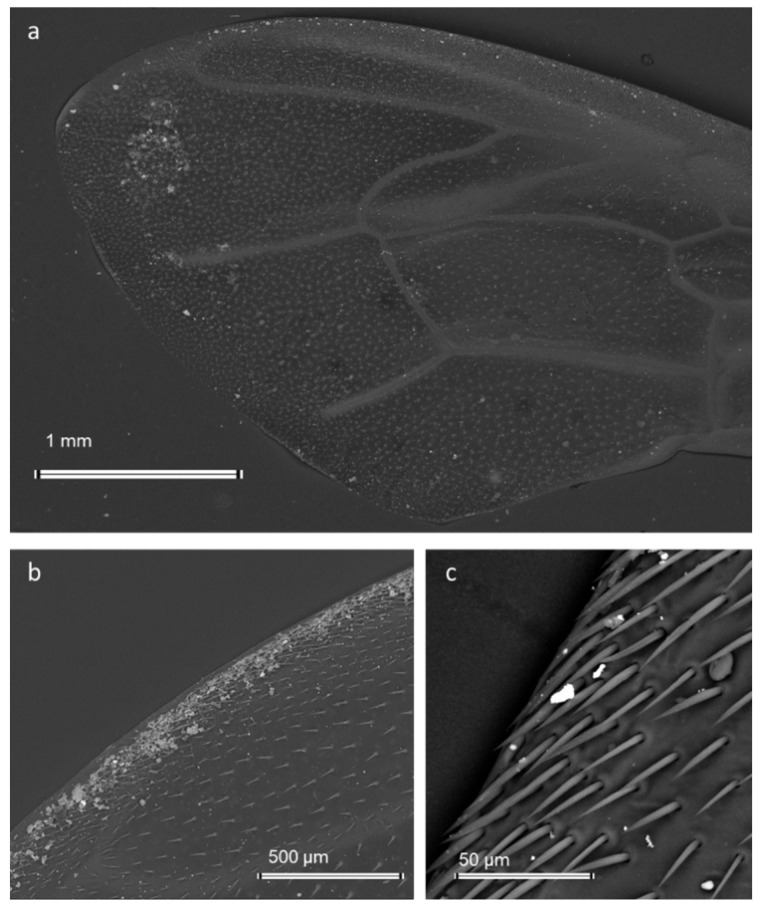
Scanning electron micrographs of the forewings of bees contaminated by airborne PM (bright and grey spots): (**a**,**c**) Backscattered electron images; (**b**) Secondary Electron image.

Due to its role as a bioindicator, the honey bee is able to provide important informations on pollutants such as heavy metals, radionuclides, and pesticides by analysing the traces present in its body and in hive products [87,88,89,90,91,92,93]. Recent studies demonstrate that forager bees are also efficient samplers of airborne PM [94,95], and especially the fraction below 10 μm in diameter [96]. During the foraging activity of nectar, pollen, and water, each bee collects samples of the main airborne PM emitted from the different sources inside the foraging area [94,95,96,97]. The foraging area can span several meters and even kilometers around the hive depending on the food resources. During flights, the hairy body of the bee accumulates an electrical charge due to the friction with air molecules and enhances the attraction with particles suspended in the air or deposited on the surfaces (soil, vegetation) on which the bee lands [94,98,99] (Figure 8).

**Figure 9:**
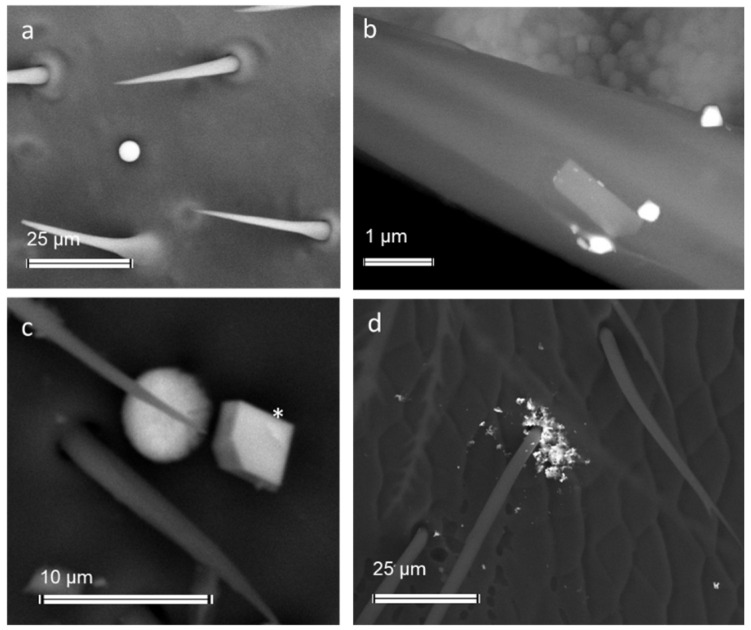
Airborne PM of anthropogenic or natural origin contaminating the body of bees: (**a**) spherical PM of iron oxides/hydroxides from a high-temperature combustion process; (**b**) fine and ultrafine PM (bright spots) of baryte from vehicular traffic on a hair; (**c**) a calcite (asterisk) of natural origin with the typical rhombohedral habitus; (**d**) fine and ultrafine PM of gold, possibly bottom ash of a waste incinerator.

Airborne PM attached to the bee body can be readily analyzed by size, morphology, and chemical composition through a Scanning electron microscope (SEM) provided with energy dispersive X-ray fluorescence (EDX) [94,95]. Coupling an efficient mobile sampler of airborne PM with a powerful technique may provide accurate identification and classification of pollutant PM and, therefore, its impact on human health [94]. In addition, single particle analysis of airborne PM collected by the bee allows for distinguishing among specific emission sources, either natural or anthropogenic. For example, studies demonstrated the specific contribution of vehicular traffic, mining operations, cement plants, waste incinerators, agricultural operations, and steel works in PM emission [94,95,96,97] (Figure 9).

Single particle counting may also provide a quantitative assessment of the relative contributions of different emission sources [97]. This, for example, allowed demonstrating that in a highly anthropized area of the Po Valley (Northern Italy), PM exposure levels vary sharply throughout the year based on recurrent local activities, for example, increases in vehicular traffic during summer holidays and local agricultural operations involving harvesting and sowing [97].

Finally, quantitative data collection and estimation of the relative contribution of the different emission sources of PM can be possible by using image-processing software. For example, bees living in a highly polluted environment showed on their body an estimated mass of 1.90 ng of pollutant PM per mm^2^, whose grain size distribution showed an exponential increase in the finer fraction [97].

### 3.3. Impact of the Exposure to Airborne PM on the Bee Health

**Figure 10:**
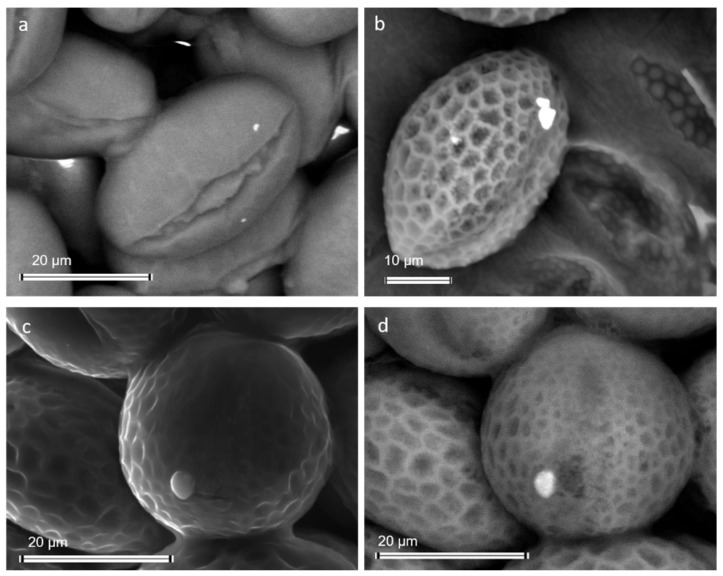
Scanning electron micrographs of bee pollen contaminated by pollutant PM: (**a**) Fine PM (bright spots) composed of iron oxides/hydroxides on alfalfa pollen; (**b**) Airborne PM of baryte (bright spots) on a pollen grain of a *Brassicacea*; Secondary (**c**) and backscattered (**d**) electron micrographs of bee pollen contaminated by silicon dioxide.

In heavily polluted environments, airborne PM can also contaminate bee products, namely honey, and pollen [100] (Figure 10). In a recent study by Papa and colleagues [96], honey produced by a bee colony living close to a highly trafficked area displayed contamination by fine and ultrafine metal-based PM derived from vehicles’ braking systems. In addition, pollen grains collected by worker bees were contaminated by the same PM [96].

This demonstrates not only the potential risk of PM entering the food chain, given that honey and pollen are widely consumed, but also a health risk for the bee itself and other pollinators. Indeed, honey and pollen represent key elements of the bee diet, securing sugars and proteins/lipids to the bee family, respectively [96]. Specific ecotoxicological studies should therefore highlight any potential risk for the health of pollinators that might be severely exposed to airborne PM with consequences on the pollination service. In the case of honey bees, the delivery to humans of many important products could also be affected. Honey bees are also insects of high economic relevance, as they may deliver honey and pollen and a wide range of products widely used in medicine, pharmacy, and cosmetics, i.e., propolis, royal jelly, wax, and venom.

Current studies on the oral exposure of pollinators to particles indicate both lethal and sub-lethal effects [101,102]. In particular, cytological and histological modifications of the gut epithelium or alterations in the gut microbial community have been demonstrated [103,104,105]. Indeed, ingested particles can come into contact with epithelial cells and the microbiome lining the gut, posing hazards to the gut community [105]. Recent evidence also suggests that gut microbiota disruption can severely affect the health of bees [106] and further studies are urgently needed to highlight any potential role of the gut microbiome alteration in the Colony Collapse Disorder, a phenomenon that causes loss of bee colonies worldwide [102].

## 4. Conclusions and Future Directions

The current knowledge and the main research lines of the updated shot of the linkage between ecosystem services, pollinators, sustainability, and health outcomes, are here given. Particularly, the direct impact on humanity was marked.

This work can provide some guidance for the formulation of protection policies on pollinators considering their fundamental contribution on ecosystem services, specifically regulating. Generally, it was evidenced how linkages between pollinators, plants, and ecological interactions lead to positive effects on ecosystem services, particularly the regulating and supporting ones. Pollinators, with several wild species, perform an ecosystem service of pivotal value considering the number of pollinators is positively correlated with number of flowers per plant [107]. A need to gather information on the threats of honeybees and other pollinators is here evident in the light of the worrying news deriving from the growing phenomenon of the decline of pollinators. The promotion of conservation, maintenance, and sustainable use of pollinators represents a key challenge. Furthermore, on the basis of very recent studies, it emerges the need to investigate in the future the factors of impact on the abundance and wealth of pollinators and the regulation mechanisms expressed in terms of variance that are not currently known [107].

On the other hand, pollinators represent valuable environmental bioindicators by also considering the increase of negative effects of air pollutants on ecosystems. In this context, the impact of the exposure to airborne particular matter was a key issue to explore, and the use of the honeybees represents an innovative tool to assess airborne particulate matter.

Airborne particulate pollutants are widespread and people living in polluted areas may be exposed for a long time to airborne particulates [96,97]. Our case study research also suggests a potential and growing risk that PM could invade the food chain and be ingested by honey bees and other pollinators. All of this suggests that for the future it is necessary to study and research ecotoxicological causes and effects to identify and quantify exposure levels and effects on human health and ecosystem services.

## Figures and Tables

**Table 1:** Role of trees to regulating services by FAO website (https://www.fao.org/ecosystem-services-biodiversity/background/regulating-services/en/). (accessed on 18 January2022).

Regulating Services	
Service	Role of trees
Pollination	Natural forests are important habitats for pollinators, providing refuge and food. Given a choice, wild honeybees chose nesting places in trees rather than in an open landscape. When enough bees are present in a forest, they provide better pollination that leads to an improved regeneration of trees and conservation of the forest’s biodiversity.
Local Climate Air Quality	Urban trees can affect air quality in the following ways: (i) converting carbon dioxide to oxygen through photosynthesis; (ii) intercepting particulate pollutants (dust, ash, pollen, and smoke) and absorbing toxic gases such as ozone, sulphur dioxide, and nitrogen dioxide; (iii) emitting various volatile organic compounds contributing to ozone formation in cities; (iv) lowering local air temperatures; (v) reducing building temperature extremes in both summer and winter and consequently reduce pollution emissions from power-generating facilities.
Carbon sequestration and storage	Trees and plants grow, thus removing carbon dioxide from the atmosphere and effectively stocking it away in their tissues.
Moderation of extreme events	Extreme weather events and natural disasters are posing an increasing threat to the world’s forests. The condition of forests themselves can influence extreme events. For example, deforestation or poor management can increase flooding and landslides during cyclones. However, the extent of large-scale flooding in the lower parts of major river basins does not seem to be linked to the degree of forest cover or the management practices in the catchment area. Similarly, forests cannot prevent large-scale landslides and mass movements triggered by tectonic or extraordinary rainfall events.
Waste-water treatment	Trees contribute heavily to waste-water treatment through their root system and their role in nutrient cycling.
Erosion prevention and maintenance of soil fertility	Studies have shown that the more closely an agricultural system resembles a natural forest in its canopy structure, tree spacing, and ground cover, the less chance of soil erosion. Traditional agroforestry techniques, which provide natural cover, have been used for centuries to produce food without causing long-term damage to the environment.
Biological control	In a forest, the biological control of pests is often the chosen methodology since the relatively stable environment of a forest guarantees freedom from such adverse effects as interference by pesticides or disturbing agricultural practices. Natural or sustainably managed forests are also a great reservoir of natural pest eradicators.
Regulation of Water Flow	Forests influence the amount of water available and the timing of water delivery. Stream-flow regulation by forests results from processes in the forest canopy, on the surface, and below the ground. Sustainable forest management is key to the regulation of water flows.

**Table 2:** Some initiatives to increase the wild bee populations and for the conservation and sustainable use of pollinators (source McGregor modified [56]).

Steps	Action
Opening up of forested areas, which created more favorable conditions for bees	Increase
Paving highways, which concentrated moisture along roadsides	Increase
Introduction of “weeds” upon which the bees forage	Increase
Growing numerous crops upon which the bees forage	Increase
Bringing desert areas into bloom (with irrigation)	Increase
Monitor pollinator decline, its causes, and impact on pollination services	Conservation
Address the lack of taxonomic information on pollinators	Conservation
Assess the economic value of pollination and the economic impact of the decline of pollinator services	Conservation
Promote conservation, restoration, and sustainable use of pollinator diversity in agriculture and related ecosystems	Conservation
